# Veterans’ Attitudes Toward Smartphone App Use for Mental Health Care: Qualitative Study of Rurality and Age Differences

**DOI:** 10.2196/10748

**Published:** 2018-08-22

**Authors:** Samantha L Connolly, Christopher J Miller, Christopher J Koenig, Kara A Zamora, Patricia B Wright, Regina L Stanley, Jeffrey M Pyne

**Affiliations:** ^1^ Center for Healthcare Organization and Implementation Research VA Boston Healthcare System Boston, MA United States; ^2^ Department of Psychiatry Harvard Medical School Boston, MA United States; ^3^ San Francisco VA Healthcare System San Francisco, CA United States; ^4^ Department of Communication Studies San Francisco State University San Francisco, CA United States; ^5^ University of Arkansas for Medical Sciences Little Rock, AR United States; ^6^ Central Arkansas Veterans Healthcare System Little Rock, AR United States

**Keywords:** smartphone apps, mobile phone, mhealth, mental health, qualitative analysis, rurality, age, veterans, depression, anxiety disorders, posttraumatic stress disorder, PTSD, alcohol abuse

## Abstract

**Background:**

Mental health smartphone apps provide support, skills, and symptom tracking on demand and come at minimal to no additional cost to patients. Although the Department of Veterans Affairs has established itself as a national leader in the creation of mental health apps, veterans’ attitudes regarding the use of these innovations are largely unknown, particularly among rural and aging populations who may benefit from increased access to care.

**Objective:**

The objective of our study was to examine veterans’ attitudes toward smartphone apps and to assess whether openness toward this technology varies by age or rurality.

**Methods:**

We conducted semistructured qualitative interviews with 66 veterans from rural and urban areas in Maine, Arkansas, and California. Eligible veterans aged 18 to 70 years had screened positive for postraumatic stress disorder (PTSD), alcohol use disorder, or major depressive disorder, but a history of mental health service utilization was not required. Interviews were digitally recorded, professionally transcribed, and coded by a research team using an established codebook. We then conducted a thematic analysis of segments pertaining to smartphone use, informed by existing theories of technology adoption.

**Results:**

Interviews revealed a marked division regarding openness to mental health smartphone apps, such that veterans either expressed strongly positive or negative views about their usage, with few participants sharing ambivalent or neutral opinions. Differences emerged between rural and urban veterans’ attitudes, with rural veterans tending to oppose app usage, describe smartphones as hard to navigate, and cite barriers such as financial limitations and connectivity issues, more so than urban populations. Moreover, rural veterans more often described smartphones as being opposed to their values. Differences did not emerge between younger and older (≥50) veterans regarding beliefs that apps could be effective or compatible with their culture and identity. However, compared with younger veterans, older veterans more often reported not owning a smartphone and described this technology as being difficult to use.

**Conclusions:**

Openness toward the use of smartphone apps in mental health treatment may vary based on rurality, and further exploration of the barriers cited by rural veterans is needed to improve access to care. In addition, findings indicate that older patients may be more open to integrating technology into their mental health care than providers might assume, although such patients may have more trouble navigating these devices and may benefit from simplified app designs or smartphone training. Given the strong opinions expressed either for or against smartphone apps, our findings suggest that apps may not be an ideal adjunctive treatment for all patients, but it is important to identify those who are open to and may greatly benefit from this technology.

## Introduction

Smartphone apps are a fast-growing mode of mental health treatment delivery with the ability to provide support, skills, and symptom tracking on demand at minimal to no additional cost to patients. Recent efforts have attempted to formally evaluate these tools, and a developing body of evidence suggests that apps can be effective in the treatment of mental health disorders [1-3]. The Department of Veterans Health Affairs (VA) has established itself as a leader in the creation of mental health apps addressing conditions ranging from depression to insomnia and posttraumatic stress disorder (PTSD). The PTSD Coach app, made available to the general public, has been downloaded over 340,000 times and has demonstrated effectiveness in multiple rigorous studies [4-7]. The rate of smartphone ownership reported by veterans ranges from 47% to 76% [8,9], and roughly 17% of veterans with PTSD have reported ever having used a health-related app [8,10], suggesting relatively low levels of current engagement.

Gaining a better understanding of veterans’ attitudes toward mental health apps may help explain their current rates of use. Survey data have provided conflicting results regarding veterans’ openness to smartphone interventions, with veterans who are currently receiving mental health treatment, perhaps, being more favorable toward this modality as opposed to those with mental health diagnoses who are not in care [8,11]. Findings from a small focus group of veterans with PTSD revealed that they were generally less comfortable navigating smartphone apps as opposed to personal computers, the internet, or email. However, several veterans reported using mental health apps, and the PTSD Coach app was found to be particularly helpful in managing symptoms and directing veterans to additional resources, such as a suicide crisis line [10].

It is particularly important to examine attitudes toward mental health app use among rural veterans, a population with lower mental health–related quality of life, greater risk of suicide, less health care service utilization, fewer specialty care services, longer travel times to clinics and hospitals, and poorer overall access to care [12-15]. This group may particularly stand to benefit from receiving mental health coping skills and support remotely via mental health apps. Smartphone ownership among rural populations, while lower on average than urban and suburban populations, continues to increase, with approximately 65% of the rural population reporting having a smartphone in 2018 [16]. While rural and urban patients were demonstrated to be equally open to integrating technology, such as telehealth appointments, into their care in one study [17], another study found rural patients to be less likely to utilize technological interventions citing attitudinal differences or network connectivity difficulties as potential explanatory factors [18]. To the best of our knowledge, openness toward the use of mental health smartphone apps and potential barriers to use have not yet been comprehensively examined among rural veterans.

Differences in attitudes toward mental health apps may also exist between older and younger veterans. Compared with their younger counterparts, older adults are often perceived as being uninterested, unwilling, or physically unable to engage with new technologies, and providers may hesitate to recommend such treatments, despite evidence demonstrating a wide range of abilities and attitudes within the aging population [19]. mental health apps could serve as an important tool within the growing population of older adults who seek health care services by increasing their sense of independence and self-management of chronic conditions [20,21]. A study of current mental health treatment seekers found older veterans to have a strong interest in mental health apps and reported a trend toward current users of PTSD apps being older, despite findings that older veterans were, on the whole, less likely to own a smartphone [8]. Similarly, large national surveys have found smartphone ownership to decline with age, although the rates of ownership continue to increase across all age groups over time [16]. Another study found that while many older adults were digitally literate, this population more often perceived technology as replacing in-person care, to which they reacted negatively [18]. Visual, motor, or cognitive impairments may make smartphone use more difficult within older populations and may decrease their confidence in navigating new technologies; these physical changes have been shown to take effect beginning at the age of 50 [19,21]. Given these varied findings, it is critical to conduct a focused examination of attitudes toward mental health app usage among younger and older veterans to better characterize the population that may benefit the most from these tools.

This qualitative study, therefore, aimed to examine attitudes toward mental health app usage among a diverse sample of rural and urban veterans who varied in age and screened positive for at least one mental health diagnosis. Semistructured interviews and qualitative analyses were conducted with the goals of examining (1) veterans’ attitudes toward smartphone apps; (2) facilitators and barriers to mental health app usage; and (3) potential differences in attitudes between rural or urban and older or younger veterans.

## Methods

### Participants

This manuscript presents a secondary analysis from a larger study examining veterans’ access to mental health care [22]. To meet the eligibility criteria, participants were required to be US military veterans ranging in age from 18 to 70 years who screened positive for PTSD, alcohol use disorder, or major depressive disorder at a VA health care appointment during the previous year, as documented in their medical record. Participants were not required to have received VA mental health services. However, veterans were excluded if they denied any distress related to the condition(s) for which they screened positive as they were deemed unlikely to require mental health treatment. In addition, veterans with psychosis or dementia diagnoses were excluded as these conditions may have limited their ability to provide informed consent and adequately complete study protocols.

### Recruitment

Participants were recruited from 9 VA community-based outpatient clinics located in Maine, Arkansas, and Northern California. To further achieve geographic diversity, at least one metropolitan facility and at least one rural facility were included within the 3 clinics sampled per state. We used a stratified purposeful sampling strategy for variability regarding age, sex, race, mental health diagnoses, and history of mental health care. Recruitment packets were mailed to 585 eligible veterans who had received health care services at any of the 9 identified VA clinics. Packets contained a letter introducing the study and explaining that the veteran could be contacted by the research team unless he or she declined participation by either calling the research office or returning a preaddressed and stamped opt-out letter. Veterans who did not opt out within 2 weeks of notification (n=496) were called by trained research staff to review study participation and confirm eligibility. Within this group, 258 veterans were reached, and 72 of them were included in the study after accounting for those who declined or were deemed ineligible. These 72 veterans, plus 8 additional veterans recruited onsite, constituted the study sample (n=80). Notably, 14 veteran transcripts contained no codes pertaining to smartphones or mobile apps (see Data Analysis section for additional information regarding coding and transcript inclusion criteria), resulting in a final sample of 66. Additional information regarding the study’s opt-out design and recruitment procedures can be found elsewhere [23].

### Procedure

Most interviews were conducted in person, and 6 interviews were completed by telephone. Participants who completed in-person interviews provided written informed consent, whereas those who participated by phone provided verbal consent. Participants then completed a battery of self-reported quantitative questionnaires followed by a semistructured qualitative interview. Interviews were conducted by a team of 4 experienced qualitative researchers, including 1 communication scientist, 1 applied anthropologist, 1 nurse scientist, and 1 clinical psychologist. Interviews lasted approximately 1.5-2 hours, and veterans received financial compensation for their participation. All research procedures were approved by the VA Central Institutional Review Board (Study #13-29).

### Measures

At the start of each interview, participants first completed a series of self-reported questionnaires related to descriptive demographics and physical and mental health histories and then completed a semistructured qualitative interview informed by the State of the Art (SOTA) Access Model [24] (Multimedia Appendix 1). The SOTA Access Model consists of 5 domains that may influence access to health care: geographical (eg, distance to a clinic), temporal (eg, appointment wait time), financial (eg, cost of care), cultural (eg, stigma surrounding mental health care), and digital (eg, ownership of a smartphone or personal computer). The semistructured qualitative interview guide was developed and pilot-tested by the study team, and it contained questions tailored to each of the 5 SOTA domains. The interviewers asked additional open-ended questions throughout to explore veterans’ experiences with access to VA health care more broadly.

### Data Analysis

We performed qualitative data analysis in 2 phases. In Phase I, the qualitative team uploaded interview transcripts into ATLAS.ti (ATLAS.ti Scientific Software Development GmbH; Berlin, Germany) for data management and analysis [25]. The team used a modified form of directed content analysis [26] to develop an original analytic codebook capturing factors related to veterans’ overall access to mental health care, including digital access such as smartphone and app use. Conducted between 2015 and 2017, this phase resulted in a large dataset of coded interviews for domains related to the SOTA Access Model and new domains described in more detail elsewhere [27,28].

In Phase 2, we examined responses within the digital domain of the SOTA Access Model and specifically focused on veterans’ discussions of and perceptions of smartphone apps as they pertain to mental health care. We used thematic analysis [29,30] to systematically identify meaningful patterns regarding veterans’ attitudes toward apps from a critical realist standpoint, meaning that participants’ statements were taken at face value, acknowledging that these interpretations are influenced by the researchers’ individual beliefs and expectations [31,32]. Analyses were informed by existing theories of technology adoption that consider the influence of multiple factors on individuals’ openness toward a novel intervention, including its perceived effectiveness, accessibility, and compatibility with ones’ values (Unified Theory of Acceptance and Use of Technology; UTAUT [33,34]). The UTAUT served as a sensitizing concept, which helped guide pattern identification and data analysis but did not prescribe the interpretation of findings [35].

To develop this secondary analysis, the lead author first read all transcripts in their entirety, noting when and how veterans discussed smartphones and mobile app use. Next, segments to which “Smartphone” and “Mobile Apps” codes within the SOTA digital domain had been applied in the overall Phase 1 dataset were identified and reread. All relevant segments were read and annotated multiple times. Then, the lead author generated and assigned subcodes to the data; the subcodes were modified and consolidated to ensure consistency and uniformity across segments. Next, patterns and relationships were identified between subcodes, resulting in the development of key themes and subthemes representing broader concepts within the data, which were subsequently reviewed by a larger team and streamlined to provide a refined summary of findings. Finally, participants’ age and urban or rural status were introduced into the analysis to assess potential patterns within themes and subthemes based on these demographic characteristics. The first author developed an analytical summary of study findings that was first discussed with the second and third authors and was then presented to the full research team for review. All revisions to the analytical summary were determined through consensus, resulting in a final model that adequately captured meaningful patterns and themes within the data.

To explore potential age-related patterns within the data, the sample was divided into older (≥50, n=25) versus younger (<50, n=41) age groups based on the literature demonstrating that cognitive and physical changes influencing technology use can begin by the age of 50 [19,21,36,37]. Rural status was determined using Rural Urban Commuting Area (RUCA) codes [38], a classification system that uses the Bureau of Census urbanized area and urban cluster definitions as well as commuting patterns to classify census tracts into 33 distinct subdivisions, which typically are consolidated into 4 categories (1) Urban areas with metropolitan cores of at least 50,000 residents and substantial commuting flow patterns to urbanized areas; (2) Large rural towns with micropolitan cores with a population of 10,000-49,999 and substantial commuting patterns to urban clusters; (3) Small rural towns with primary commuting flows to or within population centers of between 2500 and 9999 residents; and (4) Isolated rural towns, defined as less populated rural areas with no commuting flows to urbanized areas or urban clusters. We further aggregated these data based on the established RUCA recommendations to differentiate between urban and rural status (categorization method C), such that veterans falling into category 1 were designated as urban, while those in categories 2-4 were deemed rural [39].

For reporting between-group differences, we calculated the percentage of veterans within a given group (rural or urban, young or old) who made statements coded within a particular theme. Findings of group differences were reported when the percentage of respondents within a given theme was at least 1.5 times greater for the dominant group (eg, rural veterans) than that for the comparison group (eg, urban veterans).

## Results

### Descriptive Statistics

Participants within the final sample (N=66) ranged in age from 20 to 69 (mean 44.61 [SD 13.39]) years. Overall, 26% (17/66) of the sample was female and 42% (28/66) resided in rural areas. The sample was racially and ethnically diverse (47/66, 71% white; 14/66, 21% black; 7/66, 11% Hispanic; 5/66, 8% Native American or Pacific Islander; and 3/66, 5% Asian), and participants had a range of educational backgrounds, employment statuses, and incomes (Table 1). There were no significant differences between rural and urban veterans regarding age (*t*_64_=−0.50, *P*=.62) or race (*χ*^2^_1_<2.3, where *P* values for all categories were >.13), although it should be noted that all Asian respondents belonged to urban locations. Rural and urban veterans also did not differ in sex, income, employment status, or level of education (*χ*^2^_1_-*χ*^2^_9_<8.7, where *P* values for all categories were >.22). Regarding site-level differences, there were more black and fewer white participants in the Arkansas sample than in the Maine or California sample (*χ*^2^_2_>7.5, where *P* values for all categories were <.03), and all Asian participants in this study were from California (*χ*^2^_2_=8.7, *P*=.01). There were no other site-level differences regarding any demographic measures (*χ*^2^_1_-*χ*^2^_9_<3.1, where *P* values for all categories were >.21).

Veterans’ statements regarding mental health smartphone apps tended to be either strongly positive or negative in nature. We examined positive and negative attitudes within the 5 primary themes identified within the data: Treatment effectiveness, Ease of use, Culture and identity, Facilitators, and Barriers. Furthermore, we explored the potential differences in responses between rural and urban veterans, as well as between older and younger age groups; exemplar quotes as well as rurality and age differences across themes have been presented in Table 2.

### Treatment Effectiveness

#### Positive Attitudes

Overall, urban veterans made more positive statements regarding mental health app effectiveness compared with their rural counterparts. However, beliefs that mental health apps could be effective did not vary based on age. Multiple veterans who had not yet used an mental health app stated a willingness to try this new intervention and felt that apps could become an effective part of their care by serving multiple distinct functions. Some discussed how a smartphone app could act as a guide providing strategies to address or track symptoms, distract them from strong emotions at the moment, or direct them to additional resources if they are in crisis, such as a suicide hotline. Others noted the role of an app as a journal to log thoughts and feelings:

It’s also a diary of sorts. Because whatever [patients] are feeling at that time, they can just pick up their phone and put it in there, and then the therapist can go back and look at it, and be like, “This is how you were feeling this day.” It’s a nice communication tool between the two.

ID #1042, 30-year-old urban female

This participant described the potential for collaboration between the provider and patient, a concept echoed by several other veterans.

In addition to these positive statements from veterans without mental health app experience, multiple veterans reported finding apps helpful for managing mental health problems and associated symptoms. For example, several veterans noted that PTSD Coach provided effective real-time support around managing anger symptoms:

I have the PTSD app on my phone, which is really cool. It actually goes over your breathing exercises, it gives you little reminders of how to get around being angry, what to do if you find yourself in a situation where you’re going to hurt someone, it’s really helpful.

ID #2009, 44-year-old urban female

Another veteran described how quickly he was able to find relief in the moment using PTSD Coach:

I like the focusing on other stuff...you’re in an anger situation and you focus on something else and it really does work. Because it takes you out of the moment for a second and it doesn’t take long...I like how quick it works.

ID #3012, 53-year-old rural male

Although PTSD apps were most commonly mentioned, other veterans reported using sleep, relaxation, and mindful eating apps.

Some veterans noted that mental health apps could serve as a good adjunctive therapy tool, but they would not want them to replace in-person contact with their therapist:

I mean, [apps] would be helpful, but I don’t think [I would want] to have all my services done that way, versus talking to someone face to face...that would be still important when it comes to getting mental health [treatment].

ID #1037, 37-year-old urban female

**Table 1:** Sample demographics.

Characteristics		Total sample (N=66)	Urban subset (n=38)	Rural subset (n=28)
Age (years), mean (SD); range		44.61 (13.39); 20-69	43.89 (12.46); 20-69	45.57 (14.74); 26-69
Rurality (rural), n (%)		28 (42)	N/A^a^	N/A
Sex (female), n (%)		17 (26)	11 (29)	6 (21)
**Race and ethnicity, n (%)**				
	White	47 (71)	25 (66)	22 (79)
	Black	14 (21)	10 (26)	4 (14)
	Hispanic	7 (11)	6 (16)	1 (4)
	Asian	3 (5)	3 (8)	0 (0)
	Native American or Pacific Islander	5 (8)	4 (11)	1 (4)
	Multiracial	2 (3)	2 (5)	0 (0)
	Other	2 (3)	1 (3)	1 (4)
**Education, n (%)**				
	High school graduate or general equivalency diploma	12 (18)	7 (18)	5 (18)
	Technical school	1 (2)	1 (3)	0 (0)
	Some college	28 (42)	17 (45)	11 (39)
	Bachelor’s degree	15 (23)	6 (16)	9 (32)
	Master’s degree	4 (6)	4 (11)	0 (0)
**Employment status, n (%)**				
	Employed (full- or part-time)	25 (38)	15 (39)	10 (36)
	Disabled	18 (27)	12 (32)	6 (21)
	Retired	6 (9)	3 (8)	3 (11)
	Unemployed	3 (5)	2 (5)	1 (4)
	Student	5 (8)	1 (3)	4 (14)
	Other	3 (5)	1 (3)	2 (7)
**Income (US $), n (%)**				
	<10,000	9 (14)	5 (13)	4 (14)
	10,000-25,000	18 (27)	10 (26)	8 (29)
	25,001-50,000	15 (23)	9 (24)	6 (21)
	50,001-75,000	14 (21)	8 (21)	6 (21)
	≥75,001	3 (5)	2 (5)	1 (4)

^a^N/A: not applicable.

**Table 2:** Primary themes: exemplar quotes and rurality and age differences.

Theme and attitude			Exemplar quotes		Greater % of statements^a^
**Treatment effectiveness**					
	Positive	*[A mental health app] would be fantastic...whenever I have one of those outbursts and frustration, I can just open it up, say “Okay, what’s my first step?” I’m sure there’s some pamphlet or publication out there that I could use, but carrying around a pamphlet everywhere you go [is cumbersome]...*[ID #1023, 26-year-old rural male]		Urban veterans: 68% (26/38) urban 36% (10/28) rural No age differences observed	
	Negative	*[Using a mental health app] sounds crazy...I don’t think I’ve got the patience to be sitting down reading about what can help me, I’ve pretty much heard it all...it just don’t seem like it would do anything.* [ID #1004, 33-year-old rural male]		Rural veterans: 24% (9/38) urban 60% (17/28) rural No age differences observed	
**Ease of use**					
	Positive	*Everything’s really simplified with the apps. It’s easy. From my experience on the smartphone, you search almost anything, you find the one you want, you hit download. When it’s downloaded, you open it.* [ID #2002, 39-year-old urban male]		Urban, younger veterans: 45% (17/38) urban 18% (5/28) rural 39% (16/41) young 24% (6/25) old	
	Negative	*I haven’t gotten acclimated to a smartphone yet...the technology is kind of difficult to navigate through.* [ID #2023, 66-year-old rural male]		Rural, older veterans: 13% (5/38) urban 39% (11/28) rural 15% (6/41) young 40% (10/25) old	
**Culture and identity**					
	Positive	*I mean my generation, [we] don’t have that much difficulty using technology as a means of communication or seeking help, but I can imagine older veterans...they’re just not used to using technology...it might be too much change.* [ID #3003, 27-year-old urban female]		Urban veterans: 13% (5/38) urban 4% (1/28) rural No age differences observed	
	Negative	*Now I see people standing there just looking at their phone, and they’re all in a group but nobody’s talking to each otherI mean what kind of society is this...I don’t understand it, there’s no interaction with other human beings.* [ID #3019, 55-year-old urban male]		Rural veterans: 11% (4/38) urban 29% (8/28) rural No age differences observed	
Facilitators		*I went to a computer class you can sign up for while you’re inpatient [to learn] the basics, how to get on a computer. I haven’t used smartphones but...I believe I could probably use that, just pushing with your finger and all.* [ID #1005, 56-year-old urban male]		Greater % of veterans reporting mobile phone ownership were younger: 56% (23/41) young 32% (8/25) old No rurality differences observed	
Barriers		*I’ve got these [sleep] apps...but I haven’t been doing quite as much [because] for the first month [my phone] worked and then all of a sudden it did like my old phone and won’t connect to the Wi-Fi. So [I don’t] use it that much because you use up your minutes.* [ID #1006, 54-year-old urban female]		Greater % of veterans reporting not owning a mobile phone and having financial or connectivity barriers were rural and older: 16% (6/38) urban 32% (9/28) rural 12% (5/41) young 40% (10/25) old	

^a^Values indicate the raw count and percentage of veterans per group (urban or rural, young or old) who made statements within a given theme. Findings are reported as a difference between groups if there is at least a ×1.5 difference between percentages.

Several veterans expressed similar sentiments, mentioning that they were open to using apps for mood tracking or skills practice but were not interested in sharing the more personal information that they disclose in the context of in-person therapy.

#### Negative Attitudes

A considerable number of veteran statements described apps as being ineffective and unhelpful in addressing mental health concerns. These statements were more often made by rural veterans, but no clear differences emerged between age groups. One veteran noted the risk of potentially stirring up strong emotions on an app when alone:

I don’t think it would be beneficial to me to read about [my PTSD] on my smartphone. And bring up bad memories of my own and then I’m stuck right there trying to figure out how to deal with this. What do you do then?

ID #1013, 40-year-old urban male

Another younger rural veteran explained that he tends to get angry while driving and felt that it would not be feasible to turn to a smartphone app for support while behind the wheel. Some veterans described being satisfied with their current strategies for managing their mental health, including researching their condition on the internet, and, therefore, did not see an added value of using apps. Others felt that game apps or podcasts were ultimately more effective at distracting them from their negative thoughts.

Several veterans who had utilized mental health smartphone apps discussed burdensome components of these interventions. One veteran described how previously useful components of the PTSD Coach app became irritating over time as his functioning declined:

I put the PTSD app on my phone and I used it quite regularly for probably two years. And then lately I deleted it because it just got annoying. It has the constant reminders and at first it was great because it was like, aha, I can track my ups and downs...then as things progressively got worse over the past couple of years, it became a reminder of, “Hey, you do have issues...”

ID #2003, 35-year-old urban male

He went on to say that apps would be more effective if data were directly shared with clinicians as a part of his medical record so that they can be informed of changes in patient functioning and follow up during sessions.

### Ease of Use

#### Positive Attitudes

Some veterans mentioned that apps and smartphones are specifically designed to be user friendly and simple. These veterans were more often from urban versus rural locations, and younger veterans tended to discuss apps and smartphones as being easy to use more so than older veterans. Several veterans noted that having information on their phone was more streamlined and organized than using a physical journal or worksheets that could be misplaced or forgotten. One veteran described the convenience of receiving support when he needed it without having to travel and the discreetness of using an app versus having to explain why he had to leave work for an appointment. Another noted that patients may feel less anxious opening up to a device compared to a therapist:

I think it’s easier for some people to go to a computer because they may not feel judgment; the computer can’t say, “You’re doing this wrong. And you should never come and see me,” you know? So I think it’s a benefit...they may not feel as pressured and may be willing to give more information.

ID #1042, 30-year-old urban female

#### Negative Attitudes

Smartphone technologies were often characterized as being unwieldy, complicated, and mentally taxing to learn how to use. These statements were overwhelmingly made by rural versus urban veterans and by those in the older age group. Some veterans noted how technology is changing too quickly to keep up with, and others mentioned that the smartphone touchscreen was difficult to navigate. One veteran experiencing neurological symptoms described the difficulty of attempting to use a smartphone:

They can’t figure out why my hands shake so bad...so trying to use a smartphone [is frustrating]...I don’t have a whole lot of feeling in my hands...so knowing, ok I’m actually touching this thing, why is it not working? I started listening for my finger tapping the phone to see if I’m actually touching it because otherwise I can’t feel it.

ID #1013, 40-year-old urban male

Some older veterans described having trouble interacting with the small smartphone interface due to vision decline and others cited the overall burden of the aging process, as this veteran exemplified:

No can do...I don’t want to tax my brain with something like that...I’ll be 70 next year you know...my brain is just kind of wore out.

ID #3021, 69-year-old rural male

One older rural veteran with experience using the PTSD Coach app explained that it requires patients to upload their own photos and songs; he noted that this added an additional step and that he would prefer that the app was ready to use as soon as it was downloaded.

### Culture and Identity

#### Positive Attitudes

Several veterans described the compatibility of new technologies with aspects of their culture and identity. These veterans tended to be from urban locations but were distributed across age groups. One veteran noted:

I love the idea...I like carrying an iPhone...I’m a geek in some sense, I think. I kind of like messing with some of those things.

ID #2025, 65-year-old rural male

mental health apps were viewed as complementary to their lifestyle and preferences for care. Some relatively younger veterans perceived themselves as being part of a “technological generation” who are expected to embrace new innovations and were, therefore, open to the integration of smartphone technology into their health care. A considerable number of older veterans expressed either a working knowledge of smartphone technologies or an interest in honing those skills and did not cite their age as a barrier to technology adoption. One middle-aged veteran spoke about the ability of his generation to adjust to changing times:

We weren’t born into [smartphone technology].but we adapted pretty well.

ID #2005, 43-year-old urban male

#### Negative Attitudes

A theme emerged within a subset of veterans’ statements in which new technologies were found to be in broad opposition to their personal values and were viewed as a destructive force within society. More of these statements were made by rural veterans, but no patterns were observed in chronological age. Several mentioned “hating” technology and feeling forced into using it by their health care system. Some went on to say that the advent of new technologies was intended primarily for companies to make a profit at the expense of its users. A considerable number of veterans spoke of the impersonal nature of smartphone apps, as one veteran exemplified:

I really hate the times that we’re in where everything is electronic...you send somebody a text message, there’s no voice inflection, there’s nothing...there’s no feelings behind it at all, so we’re just numbing our society.

ID #2020, 28-year-old rural male

Multiple veterans expressed beliefs that technology is harming society by weakening face-to-face communication skills and limiting opportunities for human connection. Some interpreted the introduction of mental health apps as signaling the reduction or replacement of in-person therapy visits. As one veteran explained:

It’s pretty hard to make a relationship with a phone as opposed to having a face-to-face relationship with someone.

ID #2005, 43-year-old urban male

Multiple statements described the low salience of new technologies within veterans’ lives and, therefore, their low level of interest in spending time on a smartphone and utilizing its more advanced functions:

I have a little phone, a little forty-dollar phone. And phones are to talk on...and computers are to compute...now [my son] has one of those crazy little phones that you can do everything with. I just don’t have an interest.

ID #1007, 57-year-old rural female

This overall indifference toward smartphones, therefore, precluded any interest in using mental health apps.

Regarding identity, a greater number of urban versus rural veterans described being “old fashioned” or “old school” when explaining why they were not proponents of mental health apps. While most of these veterans were older than 50 years, multiple relatively younger veterans echoed this preference for older technologies. As this 27-year-old rural male explained:

I’m an old school guy, I don’t mind writing in a journal [versus using an app]...I mean I like video games like the next guy and I have a computer, but I’m not that tech savvy to be honest with you. I’m still living in dial-up. That’s where I’m at.

ID #3009

Many veterans cited generational differences when explaining who is and is not open to smartphone technologies. These statements tended to make generalizations regarding the capabilities of older cohorts of veterans:

Anybody younger than 50 has got a smartphone in their pocket, but some of these older guys don’t have a clue in life about some of that stuff...Vietnam era guys...they’re not ready for smartphone time.

ID #2025, 65-year-old rural male

In these cases, veterans inferred causal relationships between chronological age and the ability to utilize new technologies.

### Facilitators

Many veterans reported owning a smartphone or a tablet; these veterans tended to be younger in age. Those who noted prior experience using an app were overwhelmingly urban but varied in chronological age. Some reported having Wi-Fi network connectivity in their homes, which facilitated app usage by eliminating cellular data fees. Veterans often described feeling comfortable utilizing new technologies; several reported having completed degrees in computer science or related fields, and others noted frequently using smartphones as part of their job. Some veterans who were less experienced reported an interest in obtaining a device if they did not already have one or increasing their technological skills by enrolling in classes or trainings. In addition, social influence was discussed as a facilitator of app usage. One urban veteran noted that his doctor recommended apps to manage stress and monitor his heart health, while another discussed the impact of observing others utilizing health apps:

I’ve seen a lot of people, including my wife, who use iPhones. And I tried to hike the other day with a neighbor and that person has how many miles you walk and all this stuff...so many different applications. I’ve seen somebody try to lose weight with an iPhone...I think it can really be useful.

ID #3024, 53-year-old urban male

### Barriers

Some veterans reported barriers including not owning a smartphone or tablet, limited finances, or wireless connectivity difficulties; the majority of these respondents were older and from rural locations. Many veterans reported having little experience or familiarity with new technologies and had never downloaded a smartphone app. For some, the multiple steps involved in adopting smartphone technologies appeared to be more trouble than it was worth, as this veteran explained:

Don’t have the tools, don’t have the equipment, don’t have the money, don’t have the knowledge, don’t want the knowledge, don’t want to pay for that—I’d just as soon fix the house up before something goes to something else.

ID #2027, 57-year-old rural male

Several veterans reported having experience using computers or smartphones at work but noted wanting a break from technology outside of the office. They, therefore, were not interested in using apps as a component of their mental health care.

A relatively small number of veterans discussed privacy concerns as being a barrier to using mental health smartphone apps:

Smartphones are smart but we as users are not. There’s a lot of features on there that allow all these different apps and sites access to your pictures and cameras and microphone and I don’t know.

ID #3002, 35-year-old rural male

These veterans ranged in age and were predominantly from urban locations. While one veteran felt more confidence in information being protected by the VA versus outside companies, another reported mistrusting the government and was worried that the VA may expose his mental health data. One veteran noted that information relayed digitally is inherently not confidential, and another explained that it is easy and common for a mobile phone to be hacked into, which thereby limited them from sharing any personal health information on their devices.

An additional barrier to use was a lack of awareness of app availability. Multiple veterans were surprised to hear from their interviewer that the VA has developed publicly accessible smartphone apps for mental health concerns. They denied having received this information from VA providers during treatment. Several veterans stated that they planned to research these apps after the interview was complete and were curious what might be available to help their symptoms:

I never knew there was apps out there that could help with what I’m dealing with...

ID #1018, 25-year-old urban male

These veterans varied in age and were distributed between urban and rural locations.

## Discussion

### Principal Findings

We conducted a qualitative study of attitudes toward mental health app use among a diverse sample of veterans varying in age and rural status who screened positive for at least one mental health diagnosis. Veterans tended to express either a strong positive or negative stance regarding apps, and we, therefore, examined positive and negative attitudes within 5 central themes within the data: Treatment effectiveness, Ease of use, Culture and identity, Facilitators, and Barriers. We found more prominent attitudinal differences regarding rural status compared with those regarding age, such that rural veterans expressed more negative opinions regarding mental health apps than their urban counterparts, while fewer differences emerged between older and younger veterans.

Rural veterans more often expressed beliefs that mental health apps would be ineffective, difficult to use, and in opposition to their values and identity. They also reported barriers to usage more often than urban veterans, including not owning a smartphone, not having experience using apps, lacking wireless connectivity, and having financial limitations. It remains unclear to what extent rural veterans’ negativity is primarily the result of financial or infrastructural barriers versus an overall unwillingness to use mental health apps, a question that has been posed in previous research reporting similar findings [18]. Gaining a better understanding of the respective contributions of these factors will help identify points of intervention, such as offering internet or smartphone subsidies; increasing network connectivity; or providing digital literacy training to increase comfort, confidence, and openness toward new technologies [40]. As a growing number of initiatives introduce technology-based interventions into rural communities in efforts to improve access, including within the VA [41], it will be of particular importance to acknowledge and address these barriers to uptake.

Regarding age differences, the majority of those reporting not owning a smartphone were 50 years and older, and this group more often described apps and smartphones as difficult to use compared with younger veterans. However, no age differences were observed regarding beliefs that mental health apps could be effective and congruent with one’s lifestyle and values. This finding of older adults having less access to smartphones but being open to their use mirrors results within a sample of PTSD-diagnosed veterans [8] and complements findings that many older adults are digitally literate and accepting of technology-based interventions [18]. Our results oppose widely held notions that older adults are not interested in new technologies [19]; interestingly, a trend emerged within the data such that veterans made assumptions regarding older generations’ purported lack of interest in new technologies that did not prove to be true within this sample. Collectively, these misconceptions underline the need for providers to resist assuming that older patients are not interested in incorporating mental health apps into their care [8]. However, findings also emphasize that smartphones may be less accessible within older populations and that certain aspects of their design, such as their smaller typeface and touchscreen format, may prove challenging or prohibitive due to declining vision and dexterity, which may begin by the age of 50 [19,21]. Increasing the default font size, choosing apps with simpler interfaces, using tablet devices with larger screens, and bolstering confidence through smartphone training may be particularly helpful within older populations, who may lack experience successfully navigating smartphones and apps.

A central theme emerged such that mental health apps were thought to be an impersonal replacement to face-to-face time with a therapist, a perception that has been reported in prior work examining opinions toward technology-based care [18]. This finding emphasizes the need to clarify that mental health apps can serve as an adjunctive tool intended to supplement and not replace in-person psychotherapy [42]. Apps can serve as platforms to log thoughts, track symptoms, or receive psychoeducation between sessions versus the more “personal” role of a therapist. However, it is worth noting that many mental health apps are not contingent on users receiving concurrent psychotherapy services and can also serve as a helpful tool for those not receiving face-to-face mental health care.

An additional theme within the data indicated a lack of awareness about VA mental health apps among some veterans, several of whom requested additional information about this resource. Limited knowledge about app options, both among patients and providers, may be a major contributor to low observed rates of mental health app usage within veteran populations. Given that providers are typically the gatekeepers for the dissemination of new interventions, there is a need for increased provider education and training regarding mental health apps to ensure that patients are aware of available treatment options that are well-aligned with their goals and preferences [8,43,44]. As mental health apps can be useful tools regardless of whether a veteran is currently in care, information regarding app availability should also be distributed directly to veterans to further increase engagement. Furthermore, it is essential for patients to be fully informed regarding the extent of privacy provided by apps and the potential risks of logging personal information in an electronic format prior to deciding to use these tools [42]. Overall, given the strong opinions expressed either for or against smartphone apps, findings suggest that apps may not be an ideal adjunctive treatment for all veterans, but it is important to (1) identify those who are open to and may greatly benefit from this technology, (2) provide these patients with comprehensive information regarding the availability and functionality of mental health apps, and (3) tailor care to individuals’ needs, recognizing that mental health apps are one of the many treatment options available and that they may not be appropriate for every patient [9,18,19].

### Strengths and Limitations

Strengths of this study include its use of a relatively large and diverse sample regarding age, sex, race, rurality, education, and socioeconomic status. Veterans were interviewed at 9 distinct VA clinics distributed across Maine, Arkansas, and California, and the study’s qualitative analysis of in-depth interviews allowed for a thorough examination of factors that may influence openness toward mental health app use, which may ultimately help tailor interventions and improve overall access to care. Importantly, eligible veterans were not required to be seeking mental health care, which distinguishes the current findings from previous studies of exclusively treatment-seeking populations [8] or samples with experience integrating technology into their health care [10]. This allowed for a broader range of attitudes to be gathered, as the sample was not limited to those who have already demonstrated openness toward receiving mental health treatment or utilizing novel technologies.

No veterans in the sample were older than 69 years, which represents a limitation of the work. We, therefore, are unable to draw conclusions regarding more elderly populations, which would be an important extension of this study as they may demonstrate a unique set of barriers and attitudes toward mental health app use. This study also did not examine attitudinal differences based on additional demographic factors such as sex, race, income, or education. For example, previous research has found that veterans with higher levels of education are more interested in integrating technology into mental health care [11] and that women may be more likely to download a health-related smartphone app [45]. While a thorough qualitative examination of all of these factors was beyond the scope of this work, they warrant additional investigation in future research. In addition, while a strength of the sample includes its distribution across 3 distinct regions of the country, it must still be acknowledged that this represents a subset of the population and national generalizability may be limited. Moreover, the study comprised a veteran-only sample, which limits generalizability; future research should assess whether similar patterns emerge within nonveteran populations. To assess for group differences, veterans were dichotomized into old and young age groups. While this allowed for qualitative comparisons based on age, it is possible that additional information could have been gained by operationalizing age as a continuous variable. Finally, this work does not assess mental health providers’ knowledge or attitudes regarding the integration of apps into care. This is an important avenue to explore, as providers play a crucial role in disseminating information about these novel interventions and a lack of awareness on their part may have a strong influence on rates of mental health app usage.

### Conclusions

This qualitative analysis examined attitudes toward mental health app use among a diverse sample of veterans varying in age and rural status. Rural veterans expressed more negative attitudes toward apps compared with their urban counterparts. A greater number of older adults reported not owning a smartphone and found these devices more difficult to use than younger veterans, but age-related differences were not observed regarding beliefs that apps could be effective or congruent with one’s values. Our findings highlight potential areas of intervention to increase the use of mental health apps within these populations, such as by addressing financial and wireless access, digital literacy, accessibility for those with physical impairments, and dissemination of information to both patients and providers. Although mental health apps may not be an ideal treatment modality for all patients, it is important to identify the populations that may benefit from integrating these novel tools into their care.

## Appendix

Multimedia Appendix 1 Semistructured qualitative interview guide.

## Abbreviations

PTSD: posttraumatic stress disorder
RUCA: Rural Urban Commuting Area
SOTA: State of the Art
UTAUT: Unified Theory of Acceptance and Use of Technology
VA: Department of Veterans Health Affairs
