# Supplementary material for: Veterans’ Attitudes Toward Smartphone App Use for Mental Health Care: Qualitative Study of Rurality and Age Differences
Source: JMIR Mhealth Uhealth. 2018 Aug 22;6(8):e10748. doi: 10.2196/10748 (PMC6125614; doi:10.2196/10748)
Supplement: Multimedia Appendix 1 [file mhealth_v6i8e10748_app1.pdf]

Interview Guide

Introduction
Thanks for agreeing to talk with us today.  We'd like to know more about what makes it easier or harder to get the kind of help that you might need for dealing with stress-related or emotional problems. We're talking with Veterans who are currently receiving mental healthcare and also those who are receiving other kinds of care at the VA. We'd like to talk to you about your experiences or thoughts about mental health care at the VA. There are no right or wrong answers.  You are the expert here.

Semi-structured questions:

**1.** Thinking about some of the things that you just listed, tell me more about what influences Veterans when considering healthcare at the VA for emotional or stress-related problems?

**2.** Have you experienced any emotional or stress-related problems in the last year? Tell me more about this?

**3.** In the past year did you ever get help or considering getting help for [insert MH problem]?

**4.** In the past year did you consider getting help for [insert MH problem]?

**Scenario #1: If YES to getting help:**

a) Tell me more about this: Why did you get help? Where did you go?

b) Was there something that made it more difficult for you to get this care?

- *Probe for barriers to linkage and initiation of care:* location, (e.g., travel distance to provider); time (e.g., wait time); money (e.g., out-of-pocket expenses, issues of getting sick leave); cultural (e.g., trust in provider; stigmas)

c) I'd like you to talk a bit more about your experiences while getting care for [insert MH problems]. If you think about your experiences in the past year, was there anything that made it hard to stick with or keep going to [insert the kind of treatment they indicated]?

- *Probe for barriers to engagement and continuation of care*: location, (e.g., travel distance to provider); time (e.g., wait time); money (e.g., out-of-pocket expenses, issues of getting sick leave); cultural (e.g., trust in provider; stigmas)

d) How did you deal with these problems?

- *Probe to understand how they overcame any barriers to initiating and/or continuing care.*

**Scenario #2: If YES to considering care, but NO to getting help:**

a) You said that you thought about getting help at the VA, but didn't. Can you think of a specific time when this happened (i.e., thought about getting help) but decided not to? Tell me more about that.

- <u>Probe to understand what influenced decision not to get care</u>

b) Why did you decide not to go to get help at the VA?

- *<u>Probe for barriers to linkage and initiation of care</u>*

c.) And if you had decided to get help at the VA, do you think there would be anything that would make it hard to stick with or stay in care?

- *<u>Probe for barriers to engagement and continuation of care</u>*

**<u>Scenario #3: If No to considering help:</u>**

a) How come? Can you tell me a bit about why haven't you thought about getting help?

- *<u>Probe for barriers to linkage and initiation of care</u>*

b) What if you were to get care, is there anything that might make it harder for you to stick with it or stay in care?

- *<u>Probe for barriers to engagement and continuation of care</u>*

***5.*** I'd like to shift topics a bit and get your thoughts on using technology—like the internet, smart phone apps, Facebook—to get help for [insert some of the MH problems they've talked about].

***a)*** Have you ever used digital devices like smart phone apps, internet, or email to help with some of the things we've already talked about [insert MH problems they've talked about]?

***b)*** Some Veterans say that using the internet to chat or ask questions or using smart phone apps for monitoring things like mood or feelings is helpful, but sometimes they're hard to use. Based on your experiences or those of others, what do you think?

- Probe: ease of using devices; problems with downloading/uploading; access to internet, smart phones, computers

**6.** *Is there anything that I haven't asked yet that's related to our conversation that you'd like to share?*
